# Dynamic Membrane Lipid Changes in *Physcomitrium patens* Reveal Developmental and Environmental Adaptations

**DOI:** 10.3390/biology13090726

**Published:** 2024-09-16

**Authors:** Deepshila Gautam, Jyoti R. Behera, Suhas Shinde, Shivakumar D. Pattada, Mary Roth, Libin Yao, Ruth Welti, Aruna Kilaru

**Affiliations:** 1 Department of Biological Sciences, East Tennessee State University, Johnson City, TN 37614, USA; gautamd@etsu.edu (D.G.); behera@etsu.edu (J.R.B.); suhas.bio@gmail.com (S.S.); sdevaiah4@gmail.com (S.D.P.); 2 The Donald Danforth Plant Science Center, St. Louis, MO 63132, USA; 3 BioStrategies LC, 504 University Loop, Jonesboro, AR 72401, USA; 4 Kansas Lipidomics Research Center, Division of Biology, Kansas State University, 1717 Claflin Rd., Manhattan, KS 66506, USA; mrroth@ksu.edu (M.R.); lbyao@ksu.edu (L.Y.); welti@ksu.edu (R.W.)

**Keywords:** moss, fatty acids, arachidonic acid, galactolipids, gametophyte, lipid classes, phospholipids

## Abstract

**Simple Summary:**

Membrane lipid composition is crucial for growth and adaptation in organisms. This study examines the changes in membrane lipids during the development of the moss *Physcomitrium patens*. We found that during vegetative stages, especially in the protonema, the moss exhibits high lipid content and a significant presence of very long-chain polyunsaturated fatty acids, like arachidonic acid (C20:4). These lipids, not found in vascular plants, likely help mosses adapt to cold climates and other stresses by maintaining membrane fluidity. Additionally, galactolipids such as monogalactosyldiacylglycerol are abundant, supporting chloroplast formation. As the moss transitions to the sporophyte stage, lipid composition shifts. Galactolipids decrease, while phospholipids, particularly phosphatidylcholine (PC) and phosphatidic acid increase. These changes are linked to stress protection and gametangia formation. The increased PC relative to phosphatidylethanolamine in sporophytes suggests a protective mechanism against environmental stresses. Our findings highlight the importance of membrane lipids in moss development and adaptation. Future research should explore the role of lipids in stress responses and evolutionary adaptations in bryophytes, offering insights into plant lipid biology and evolution.

**Abstract:**

Membrane lipid composition is critical for an organism’s growth, adaptation, and functionality. Mosses, as early non-vascular land colonizers, show significant adaptations and changes, but their dynamic membrane lipid alterations remain unexplored. Here, we investigated the temporal changes in membrane lipid composition of the moss *Physcomitrium patens* during five developmental stages and analyzed the acyl content and composition of the lipids. We observed a gradual decrease in total lipid content from the filamentous protonema stage to the reproductive sporophytes. Notably, we found significant levels of very long-chain polyunsaturated fatty acids, particularly arachidonic acid (C20:4), which are not reported in vascular plants and may aid mosses in cold and abiotic stress adaptation. During vegetative stages, we noted high levels of galactolipids, especially monogalactosyldiacylglycerol, associated with chloroplast biogenesis. In contrast, sporophytes displayed reduced galactolipids and elevated phosphatidylcholine and phosphatidic acid, which are linked to membrane integrity and environmental stress protection. Additionally, we observed a gradual decline in the average double bond index across all lipid classes from the protonema stage to the gametophyte stage. Overall, our findings highlight the dynamic nature of membrane lipid composition during moss development, which might contribute to its adaptation to diverse growth conditions, reproductive processes, and environmental challenges.

## 1. Introduction

Cell membranes primarily constitute of lipids that maintain their fluidity and permeability [1,2,3]. The plasma membrane serves as a permeability barrier, while intracellular organelle membranes define compartments and provide supporting matrices for various catalytic processes. Membrane lipids also serve as precursors for various macromolecule syntheses and signaling molecules [4]. Lipid composition is dynamic and varies across all organisms, among tissues, cells, and organelles, as well as through the life cycle and in response to environmental conditions. The ability to extensively remodel membranes to adopt diverse lipid compositions is an evolutionary feature that can influence the emergence of traits and species. For example, the evolutionary success and resilience of bryophytes to various stressors is in part attributed to their unique lipid components [5,6,7,8,9].

Bryophytes are among the first nonvascular plants to make a successful transition from water to land about 500 million years ago (Mya). Mosses share common ancestry with flowering plants but diverged early during land plant evolution, around 200–400 Mya [10,11,12]. At least 66% of the transcribed genes in the moss, *Physcomitrium patens*, are homologues of genes in *Arabidopsis thaliana* [13]; the similarities include genes involved in lipid metabolism. Despite this resemblance, bryophytes typically possess a distinct lipid makeup [5,14]. Membrane lipids of mosses include glyco-, phospho-, and neutral lipids. Glycolipids predominantly include monogalactosyldiacylglycerol (MGDG) and digalactosyldiacylglycerol (DGDG), which are plastidial lipids produced by the prokaryotic lipid biosynthetic pathway [15]. Phospholipids are predominant in most extraplastidic membranes, including the mitochondrial envelope, and are produced by the eukaryotic pathway [16]. Phospholipids vary in the head groups attached to the phosphate and include phosphatidylcholine (PC), phosphatidylethanolamine (PE), phosphatidylserine (PS), phosphatidylinositol (PI) phosphatidic acid (PA) and phosphatidylglycerol (PG). In addition to the head group (glyco or phospho), each of these lipid classes also contain two acyl chains esterified to the glycerol backbone (*sn1* and *sn2*). The acyl chains define the molecular species of the lipid and can differ in their length and number of double bonds. 

Mosses have copious amounts of very long-chain polyunsaturated fatty acids (VLC-PUFAs, >C_20_) in the membrane, especially a high abundance of arachidonic acid (AA; 20:4), which is typically absent in angiosperms [14,17]. Additionally, like seed plants, they contain saturated FA (16:0) and unsaturated long-chain FA (16:3, 18:2 and 18:3) in their glycolipids and phospholipids [14,17,18]. While the VLC-PUFAs are prevalent in marine organisms such as microalgae [19,20], fish, and mammals, the seed plants typically have primarily 16–18C fatty acyl species, and any longer chain lipids usually have only one or two double bonds. Thus, the prevalence of VLC-PUFA with 4 or 5 double bonds in mosses and their absence in vascular plants suggests evolution of distinct lipid metabolism and regulation between vascular and nonvascular plants [8,17]. 

**Figure 1:**
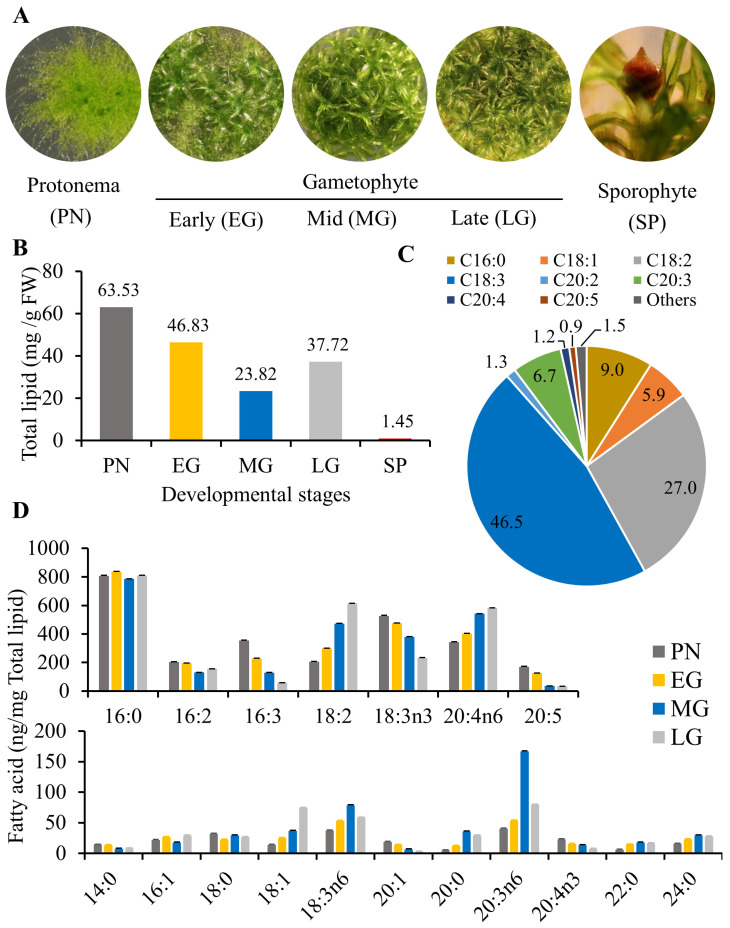
Fatty acid profile of different developmental stages in *P. patens*. (**A**) Visual representation of five developmental stages of the moss; protonema (PN), early gametophore (EG), mid-gametophore (MG), late gametophore (LG) and sporophyte (SP); (**B**) Total lipid content of each developmental stage in mg/g fresh weight (FW) of tissue; (**C**) Distribution of fatty acids (FAs) in the sporophyte; (**D**) Major fatty acid (top) and minor fatty acid levels in *P. patens* during developmental stages.

Mosses, with their distinct FA composition and unique position in the plant kingdom, serve as an excellent model system to study evolution of lipid-mediated functional divergence. Among bryophytes, *P. patens* is a well-studied organism [21] since its genome has been sequenced [22]. An additional advantage is that its life cycle is completed in 3–4 months [23], and it can reproduce sexually and asexually [24]. Typically, a dominant haploid (n) gametophyte phase is maintained under their normal growth temperatures (20–22 °C) through vegetative propagation. Sexual reproduction involves a short, diploid (2n) sporophyte phase that releases haploid spores at maturity. In *P. patens*, the development of male and female gametangia on the same gametophore [14,25] leads to self-fertilization in moist conditions. At reduced temperature (15 °C), a diploid zygote develops into a mature sporophyte [13,26]. Germination of spores gives rise to haploid filamentous protonema, which in turn give rise to mature gametophores (or phyllids). The alternation of generations in the moss life cycle includes a dominant haploid (gametophyte) phase with short-lived protonema and prolonged gametophore (early, mid, and late) stages and the transient diploid (sporophyte) stage (Figure 1A). 

Although *P. patens* is an established model system and is known to have unique lipid and fatty acid composition, lipid composition and distribution in various developmental stages and associated physiological role is understudied. Membrane lipids play a vital role in the adaptation of mosses to environmental changes during development [5]. Thus, considering the duration and environmental conditions necessary for the completion of the *P. patens* life cycle, we hypothesized that specific changes in lipid classes and their molecular species are associated with development and are likely distinct from other eukaryotes. We used electrospray ionization tandem mass spectrometry (ESI-MS/MS) to identify and quantify various lipid classes and their molecular species in five developmental stages of the moss. Additionally, we analyzed the lipids of green tissues of the lycophyte (club moss), *Selaginella moellendorffii* to draw comparisons with the moss lipids, offering insights into early vascular plant lipid profiles. We also compared the data on moss lipids with previously published lipid data of *Arabidopsis* seedlings and seeds [27] and brain tissue of mouse [28] to provide context for the 20:4-containing moss lipids, since 20:4 plays important roles in mammalian signaling, including acting as a precursor to anandamide, a signaling molecule in the endocannabinoid pathway. Understanding dynamic changes in lipid content and composition is crucial to appreciating their evolutionary role in membrane and cell functions, development and growth, and plant stress responses.

## 2. Materials and Methods

### 2.1. Plant Material

The moss *Physcomitrium patens* (sub-species *patens,* ecotype Gransden 2004) [22] was kindly provided by Drs. Quatrano and Perroud, Department of Biology, Washington University, St. Louis, MO, USA. For tissue culture, gametophores were routinely maintained on BCD agar media (1 mM MgSO_4_, 1.8 mM KH_2_PO_4_ [pH 6.5], 10 mM KNO_3_, 45 µM FeSO_4_, 8 g/L agar) containing inorganic salts (1 mM CaCl_2_) and a nitrogen source as previously described [29,30,31]. For vegetative propagation, single gametophores were transplanted into freshly prepared and sterilized BCD plates using sterilized forceps. For protonema culture, 4–6 week-old gametophores were homogenized and grown on BCD media supplemented with 5 mM ammonium tartrate (AT) and overlaid with a cellophane membrane [31]. All plates were wrapped with micropore tape and kept in a long-day growth chamber (16 h/8 h L/D at 24 °C, 60% RH, and light intensity: 40 μmol photons m^−2^ s^−1^). 

### 2.2. Sporophyte Production and Isolation 

Sporophytes were collected from gametophores grown on peat pellets in magenta boxes. To propagate gametophytes on peat pellets, Jiffy-7 peat pellets (Jiffy Products International AS, Kristiansand, Norway) were soaked in distilled water and each pellet was placed in a GA7 magenta box (Magenta Corporation, Chicago, IL, USA). A piece of muslin cloth (~4 cm^2^) was spread on top of the peat pellet and the magenta boxes were closed and autoclaved. After cooling, ~30 mL of sterile distilled water was added to each box. Homogenized protonema suspension (1 mL) was carefully inoculated on sterilized muslin cloth spread on top of water-soaked peat pellets. Moss cultures on peat pellets were then incubated at 15 °C under a 16-h light/8-h dark cycle. When moss is grown on the peat pellets in magenta boxes, the fertilization occurs naturally due to water saturation. After 8–10 weeks, the mature brown sporophytes were harvested with fine forceps from the gametophyte, followed by snap freezing in liquid N_2_ and storage at −80 °C until use. 

### 2.3. Extraction of Lipids

For lipid extraction, 7-day-old protonema, gametophores after 15 (early), 30 (mid), and 60 (late) days from the day of culture and mature sporophytes were harvested, their fresh weights were measured and stored in −80 °C freezer until further use. An initial supply of sporophytes for establishment of protocols was provided by Dr. Stefan Rensing, University of Freiburg, Germany. Young leaf tissues from two-month-old *Selaginella moellendorffii* plants (purchased from Plant Delights Nursery, Raleigh, NC, USA) were also used for lipid extraction. 

Lipid extraction was done as previously described [32]. About 200 mg of tissue from each developmental stages of *P. patens* and *Selaginella* were weighed into individual screw-capped glass tubes. The tissue samples were homogenized by vigorously vortexing in 2 mL hot isopropanol (70 °C) with ~10 steel beads (3 mm). A bead beater was used to pulverize the sample. Samples were placed in a water bath preset at 70 °C for 5 min to complete lipase inactivation. One mL of chloroform and 250 μL water were added to obtain a ratio of 2:1:0.45 mL isopropanol: chloroform: water. The samples were vigorously vortexed again and stored overnight at 4 °C. The next day, the samples were vortexed again and centrifuged for 5 min at 5000× *g*. The supernatant was transferred to a test tube, and 1 mL of chloroform and 2 mL of 1 M KCl were added to bring about phase separation. The samples were vortexed and centrifuged and the top aqueous and interphase were aspirated using a glass pipette. Samples were washed using 2 mL of 1 M KCl. The organic phase was separated and dried using nitrogen gas. The lipid samples after drying were dissolved in 1 mL of chloroform and transferred to pre-weighed glass vials. The chloroform in the lipid samples was evaporated under nitrogen gas. Total lipid weight was determined gravimetrically by weighing each glass vial with dried lipid and subtracting the weight of the empty glass vial. Total lipids were shipped on dry ice to Kansas Lipidomics Research Center for fatty acid and polar lipid analyses. Upon arrival, each sample was dissolved in 1 mL chloroform, and 3 to 36 μL of each sample were used for polar lipid analyses.

### 2.4. Polar Lipid Analyses

Polar lipids were analyzed by electrospray ionization triple quadrupole mass spectrometry (ESI-MS/MS; API4000, Sciex, Framingham, MA, USA) in direct infusion mode, and data were processed and normalized to internal standards, as described by Shiva et al. (2013) [33]. Scan modes for identification of lipid classes are shown in Supplementary Table S1. Four to six biological replicates of each plant stage were processed and analyzed. The galactolipid data were corrected for the response factor of the mass spectrometer for the analytes in comparison to the internal standards. The response factors were determined by comparison of ESI-MS/MS signals of standards and analytes quantified by gas chromatographic analysis of fatty acid methyl ester (FAME) derivatives. Q-test for discordant data was performed on the replicates of the total lipid [34]. 

### 2.5. Analysis of Fatty Acid Composition

Gas chromatography was utilized to measure the fatty acid composition of moss total lipids in the form of their fatty acid methyl ester (FAME) derivatives. For each plant stage, 100 μL of each sample were pooled and 50 nmol pentadecanoic acid (15:0; internal standard) was added, and the chloroform evaporated under nitrogen vapor. After adding 1 mL of 3 M methanolic HCl and bubbling with nitrogen, the samples were kept at 78 °C for 30 min. After cooling down to room temperature, 2 mL water and 2 mL hexane:chloroform (4:1) were added, and the samples were vortexed and centrifuged to separate the phases. The upper organic phase containing the FAMEs was transferred to a new tube. An additional extraction with 2 mL hexane:chloroform (4:1) was performed, and the upper phases combined. The organic solvent was evaporated under nitrogen, and the methyl esters were dissolved in 100 μL hexane and transferred to a GC vial. Three technical replicates per sample were prepared.

For GC-flame ionization detection, 1 μL of each technical replicate was injected in the splitless mode to an Agilent 6890N GC (Agilent Technologies, Santa Clara, CA, USA) coupled to a flame ionization detector and fitted with a 60-m DB-23 capillary column (internal diameter, 250 µm; film thickness, 0.25 µm). Helium was the carrier gas at a flow rate of 1.5 mL/min. The back inlet was operating at a pressure of 36.01 psi and 250 °C. The GC initial oven temperature was 150 °C for 1 min, followed by an increase at 25 °C/min to 175 °C and 4 °C/min to 230 °C, followed by a hold at 230 °C for 8 min. The detector was operated at 260 °C. The hydrogen flow to the detector was 30 mL/min and air flow was 400 mL/min. The sampling rate of the flame ionization detector was 20 Hz. Data were processed using ChemStation (version E. 02. 02).

For GC-mass spectrometry (MS), 1 μL of each sample was injected in the splitless mode to an Agilent 6890N GC coupled to an Agilent 5975N quadrupole mass selective detector operated in the electron impact mode at 70 eV ionization energy. The GC was fitted with a 60-m DB-5MS capillary column with a 5% phenyl, 95% methylpolysiloxane stationary phase (internal diameter, 250 µm; film thickness, 0.25 µm). Helium was the carrier gas at a column flow rate of 1 mL/min. The front inlet was operating at a pressure of 24.93 psi and 280 °C. The GC oven was held at 150 °C for 1 min, followed in an increase at 5 °C/min to 300 °C, and a hold at 300 °C for 5 min. The MS quad temperature was 150 °C and the MS source temperature was 230 °C. Data acquisition was in scan mode from *m*/*z* 50 to 650. Data were processed using ChemStation.

### 2.6. Double Bond Index (DBI Calculation)

The DBI for each lipid class was calculated as

$$\begin{array}{l} {}[\mathrm{Sum}\;\mathrm{of}\;(n\times \%\;of\;total\;lipids)\;\mathrm{for}\;\mathrm{each}\;\mathrm{molecular}\;\mathrm{species}\;\mathrm{in}\;a\;\mathrm{class}]÷\\ \left(\%\;of\;total\;lipids\;in\;the\;class\times number\;of\;acyl\;chains\;per\;molecule\;in\;that\;class\right) \end{array}$$

where n=the number of double bonds in all acyl chains in a molecular species.

### 2.7. Data Analyses

Data for each sample include 4–6 biological replicates and are expressed as mean ± SD. One-way analysis of variance (ANOVA) between the developmental stages was carried out with pairwise Tukey’s test using Minitab statistical software(Minitab18). For comparative analyses, values of moss samples were compared with those of *Selaginella* and previously analyzed seed and 8-d seedlings of *Arabidopsis* [27] and also with brain tissue of mouse, when relevant [28].

## 3. Results and Discussion

### 3.1. Membrane Lipid Composition Varies Dynamically between Physcomitrium Vegetative and Reproductive Stages

Understanding the dynamic nature of membrane lipid composition in mosses is important since their growth conditions vary dramatically throughout their life cycle. The haploid gametophyte stage is maintained at 22 ± 2 °C and the sporophyte stage is initiated only at around 15 °C. Fatty acid synthesis precedes membrane lipid synthesis, and fatty acid saturation levels determine the membrane stability and fluidity. Therefore, we evaluated the total acyl content and composition of the moss total lipids in five developmental stages (Figure 1A). The filamentous protonema has the highest total lipid content (63.53 mg/g FW) among all the vegetative stages (Figure 1B). This juvenile stage is marked by the presence of sub-apical chloronema cells that harbor numerous chloroplasts and the apical caulonema that has fewer chloroplasts [35,36]. Active cell division of both cell types demands high synthesis of membrane lipids. Some of the protonema cells differentiate to shoot-like phyllids and root-like rhizoids, which precede the relatively slow-growing gametophyte. The reproductive phase, i.e., the sporophyte, has only 1.45 mg lipid/g FW, which is 45-fold lower compared to the protonema, and 26-fold lower than the late gametophore (Figure 1B). Since the sporophyte is non-photosynthetic, gene expression related to carbohydrate metabolism is greatly reduced during the gametophyte-to-sporophyte phase transition, accounting for 25% of all differentially expressed genes [37]. As the metabolic product of photosynthesis serves as the precursor for lipid biosynthesis, a reduction in total lipid content is expected in the sporophyte compared to the gametophyte. Similarly, transcripts related to lipid metabolic processes are reduced during the gametophyte to early and mid-sporophyte phase transitions, accounting for 23.2% and 19.2% of all differentially expressed genes, respectively [37].

Unlike vascular plants, mosses contain substantial amounts of VLC-PUFA, including arachidonic acid (AA; 20:4) (Figure 1C,D) [17,38]. In gametophytes, AA accounts for 13–22% of all fatty acyl species present in the total lipid, while most of the FA species are 16:0, 18:2, 18:3 (Figure 1D). In the gametophytes 18:1, 18:2, 20:4n6, 22:0, 20:0, and 24:0 increased during maturation while 18:3, 20:5, 20:4n3, and 20:1 declined (Figure 1D). Contrarily, sporophyte had greatly reduced AA content (1.2%), while 18:3 (~46%), and 18:2 (27%) represented the bulk of FA species (Figure 1C). Although only 5% of all the fatty acids in the *Arabidopsis* leaf tissue are VLCFA [39], in *P. patens*, >25% of their acyl species are VLCFAs (20:3, 20:4, 20:5, 20:0, 20:1, 22:0, 24:0), with AA being the most abundant. In the plant kingdom, the occurrence of VLC-PUFAs is limited to non-vascular plants, and their relevance is unclear. Arachidonic acid is the precursor for eicosanoids that play an important role in inflammatory processes in mammals [40]. In mammals, an ethanolamide derivative of AA, anandamide, serves as ligand for endocannabinoid signaling [41,42,43,44] that regulates several neurological and physiological functions [44,45,46], and recent studies showed the occurrence of ethanolamides of the corresponding VLC-PUFAs, including anandamide in moss [8,47]. However, the *P. patens* mutants, incapable of synthesizing AA, did not show any visible phenotype both in normal and stressed growth conditions, posing questions on the physiological role of AA [48,49,50,51]. Although the presence of AA in moss is attributed to be a remnant feature of their marine ancestry [14], its exact role in terrestrial adaptation is yet to be elucidated.

### 3.2. Sporophytes Have an Altered Galactolipid Composition

**Figure 2:**
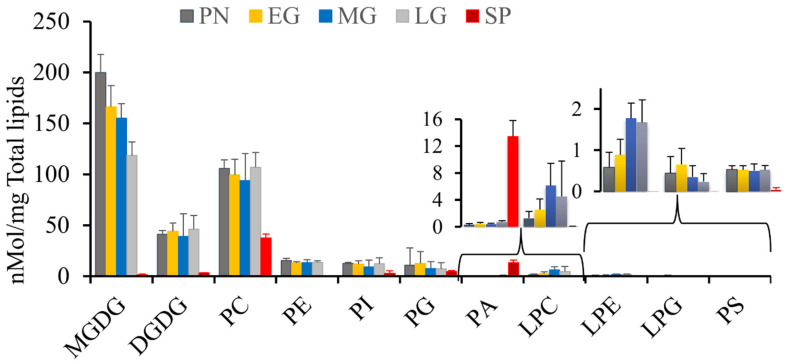
Major and minor lipid classes of the moss. Lipid content in various lipid classes at different developmental stages; protonema (PN), early gametophore (EG), mid-gametophore (MG), late gametophore (LG) and sporophyte (SP).

**Figure 3:**
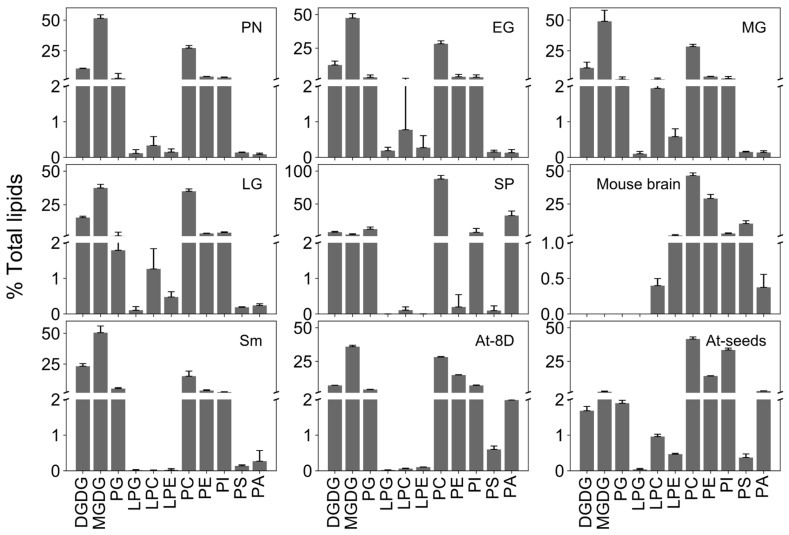
Distribution of lipid classes. The percentage of each lipid class is shown for various developmental stages of the moss (protonema (PN), early gametophore (EG), mid-gametophore (MG), late gametophore (LG) and sporophyte (SP)), Selaginella (Sm), Arabidopsis (At) seedlings and seeds [27], and mouse [28]. The graphs indicate percentage of the total lipid weight for each lipid class.

To further understand the association between membrane lipid composition and developmental changes, we quantified 11 lipid classes and their molecular species. Based on their abundance, we categorized them to major lipids: Monogalactosyl diacylglycerol (MGDG), digalactosyl diacylglycerol (DGDG), phosphatidylethanolamine (PE), and phosphatidylcholine (PC), along with minor lipids such as phosphatidylserine (PS), phosphatidic acid (PA), phosphatidylinositol (PI), phosphatidylglycerol (PG), lysophosphatidylethanolamine (LPE), lysophosphatidylcholine (LPC), and lysophosphatidylglycerol (LPG) (Supplementary Table S2, ESI-MS/MS lipid data). The levels of galactolipids were abundant in all vegetative stages, with MGDG being predominant (Figure 2). The total MGDG in vegetative stages was similar, except for the late gametophore, which produces only 59% as much MGDG as the protonema (Supplementary Table S2). There was no significant difference in the abundance of DGDG across all vegetative stages (Figure 2). Sporophytes contained much lower levels of galactolipids (Figure 2; Supplementary Table S2), comparable to those of *Arabidopsis* seeds (Figure 3). In contrast, *Sellaginella* leaves, and *Arabidopsis* seedlings displayed higher levels of galactolipids, comparable to those of moss vegetative tissues, i.e., the protonema and gametophore stages (Figure 3).

When the lipids are considered in percent composition, early vegetative stages of the moss also have more galactolipids, compared to late gametophore, and declined much more in the sporophyte stage. 

The relative proportion of MGDG in total lipids account for 52%, 38%, and 3% in protonema, late gametophore, and sporophyte, respectively (Figure 3). DGDG contributed to 11% of total lipids in protonema, increased to 15% in late gametophore, and declined to 5% in sporophyte (Figure 3). These changes in proportion of galactolipids imply that during early developmental stages, chloroplast biogenesis is occurring rapidly, and cells required higher level of galactolipids, especially MGDG. The low percentage of galactolipids in the sporophyte could be the result of decreased biogenesis of chloroplasts and the photosynthetic apparatus and a decrease in the rate of cell division and growth [52]. MGDG promotes membrane stacking, stabilizes the inner membrane leaflet in grana discs, and conserves energy produced from photosynthesis. A higher content of MGDG is required for maintaining membrane permeability and thermal stability of photosystem II (PSII) [53]. DGDG is also a structural constituent of PSII and is important to maintaining its optimal function [54]. The ratio of DGDG to MGDG (D/M) is often altered when plants are under stress. Abiotic stressors such as cold and drought increase the D/M in plants to enhance the membrane stability [55,56]. A higher D/M is needed for correct protein folding, trafficking, and protein insertion in the chloroplast envelope [57,58,59]. Our results indicate a low DGDG to MGDG ratio (D/M) in all vegetative growth stages, which increased approximately 5-fold in sporophytes, indicating that sporophytes have higher DGDG compared to MGDG. During freezing and drought stress, MGDG can be converted into DGDG by SENSITIVE TO FREEZING 2 (SFR2), resulting in DGDG that is stereochemically distinct from normal DGDG, though not differentiated by MS [60]. Additionally, during phosphate deprivation, extraplastidic phospholipids are degraded and replaced with galactolipids, particularly DGDG, which is sent outside the plastids to replace phospholipids, thus not altering the D/M ratio within the plastid [60]. Phospholipids could be degraded and converted into plastidic lipids either through phospholipase D-mediated degradation or using PA phosphatase to produce DGDG [54]. This mechanism may occur during the transition from the vegetative to reproductive phase in moss, potentially increasing reproductive success by enhancing the stress tolerance of reproductive tissues.

### 3.3. Phospholipids Are Abundant among the Major Lipids in Sporophyte 

Among the remaining major lipids, PC was the most abundant throughout the moss life cycle. Although there was not much variation in the early vegetative stages, the PC content was similar in the various gametophore stages and then decreased significantly to 37.5 nmol/mg in the sporophyte (Figure 2; Supplementary Table S2). Phospholipids majorly contributed to total lipids in the sporophyte with the PC alone comprising of 59% of the total lipids. PC was the predominant membrane lipid in non-green tissues, and this was comparable to *Arabidopsis* seeds where PC comprises 42% of total lipids and mouse brain with 46.5% PC (Figure 3). On the other hand, the PE content remained unchanged throughout development except for a sharp reduction in sporophyte (Figure 2) to 0.079 nmol/mg (Supplementary Table S2). PE in moss vegetative stages is 4% of total lipids, which is similar to that in the *Sellaginella* (4%) but distinct from the *Arabidopsis* seedlings (15%) (Figure 3). The sporophyte has only 0.1% PE of total lipids compared to 14% in *Arabidopsis* seeds. Thus, the ratio of PC:PE is higher in the moss; specifically in the sporophyte, in which the ratio is 472. The outer leaflet of the plasma membrane consists of PC and respond to environmental stimuli, whereas PE is present primarily in the inner leaflet [61]. Because PC is a bilayer stabilizing lipid [62], it acts as protective barrier in response to stress [63]. A higher ratio of PC:PE can be an indication of cold temperature adaptation in sporophytes which develop upon a drop in temperature. Further, higher PC:PE were reported in phospholipase D (PLD) deficient *Arabidopsis* when compared with WT, during freezing stress [64]. The high levels of PC suggest that PLD-mediated hydrolysis of PC is minimal in the sporophyte. PC is generally located in the envelope membranes of plastids and contributes to overall membrane stability. While its role in protecting the thylakoid membrane, which contains PSII, is minimal, its protective ability can change with alterations in FA composition [63]. Therefore, the accumulation of PC in moss could play a role in maintaining envelope membrane stability during various developmental stages exposed to different environmental conditions.

### 3.4. Sporophyte Showed a Distinct Minor Lipid Profile

The minor lipids in all the developmental stages are mostly composed of phospholipids (Figure 2). Among them, phosphatidic acid (PA) was predominant in sporophytes; PA generally accumulates in response to various stressors in plants [65]. Specifically, PA was 13.46 nmol/mg, accounting for 21% of total lipids and being the second most abundant lipid class in sporophytes (Figure 3). Among the vegetative stages of the moss, there was no significant difference in PA, with very low levels present (Figure 2). Similarly, *Sellaginella* (0.63 nmol/mg) and *Arabidopsis* seedlings (10.22 nmol/mg) have very low PA contents (Figure 3). The percentage of PA increased in the moss during transition of late gametophore to sporophyte. PA is one of the simplest form of lipids and can be generated through by PLD-mediated hydrolysis of other phospholipids in stress conditions [66,67,68,69]. Hence, the elevated levels could be a response to low temperature exposure and a signal for gametangia formation and a response to transition into cold temperature. Similarly, the accumulation of PA in *Arabidopsis* was also reported in response to low temperature [70] and freezing stress [64]. It also acts as an intracellular messenger to induce cellular responses, interacts with other proteins to induce transcription and affects hormone signaling in plants [71]. Although its exact role in reproductive phase transition is unknown, it is reported to accumulate at higher levels in reproductive organs [72] and plays a major role in tube elongation and germination of pollen [73]. In stamens and pistils of *Petunia hybrida*, PA levels were higher than that in petals or leaves [72]. Similarly in *Arabidopsis*, PA levels were highly elevated in *ap3-3* homeotic mutant, which converts petals to sepals and stamens to pistil, compared to WT [74]. Also, pollen germination and tube growth are reported to be stimulated by the increasing levels of PA [73]. Thus, the higher percentage of PA in sporophytes may be induced by low temperature cues required for sporophyte formation. On the other hand, PA is an intermediate in the glycerolipid metabolism in plants as its interconversion to diacylglycerol (DAG) through dephosphorylation is utilized for storage oil (triacylglycerol; TAG) biosynthesis. Since bryophyte spores accumulate a considerable amount (4.1–29.3%) of storage lipids by dry mass [75], it is possible that the PA detected in our study is an intermediate substrate for TAG synthesis during spore formation. 

PG was most abundant in protonema and early gametophores, decreasing by 64% in sporophytes compared to the late gametophore stage. Interestingly, while PG constitutes 2–4% of the total lipids in vegetative stages, it accounts for up to 7% in sporophytes (Figure 3). Similar to MGDG, PG is synthesized in the chloroplast, and is required for photosynthesis in plants and cyanobacteria, and regulates growth and development [76]. In *Synechocystis* sp mutants, external PG was able to rescue the growth of cells [77]. In *Arabidopsis*, PG plays an important role in development of thylakoid membranes [78,79]. Hence, a relatively higher PG content in the vegetative stages of moss ensures normal chloroplast development. 

PS was present in minor amounts in vegetative stages (~0.5 nmol/mg) which declined significantly in the sporophyte (0.039 nmol/mg). Similarly, *Arabidopsis* seedlings have 22.2-fold higher PS compared to seeds. PS does not accumulate to high levels, as it can metabolized to PE. Phosphatidylserine decarboxylase which is present on the mitochondrial inner membrane can convert PS to PE [80]. This could be a possible reason why PE levels are higher compared to PS in vegetative stages and in the sporophyte of the moss (Supplementary Table S2). Among lysophospholipids, LPE, LPG, and LPC were present in higher levels in the vegetative stages with only very small amounts of LPC detectable in sporophytes. The average content of LPC, LPE, and LPG in vegetative stages of the moss was higher than that in *Arabidopsis* seedlings and *Sellaginella*. Previous studies have suggested that lysolipids accumulate in response to various factors, including freezing in *Arabidopsis* [64], wounding in tomato plants [81,82], and pathogen infections [83,84]. Lysolipids were also detected as biomarkers of cold stress in *Stephanodiscus* sp. [85]. The high levels of lysolipids in the moss as compared to vascular plants could indicate the role of lysolipids in stress tolerance.

### 3.5. Reproductive Phase Transition Is Marked by Reduced 40C Acyl Species 

Considering the differences in major and minor lipids and the changes observed with development, we further aimed to identify whether acyl composition and levels of saturation vary with development. Therefore, we analyzed molecular species for four major lipid classes MGDG, DGDG, PC and PE, and also compared them with *Sellaginella*, *Arabidopsis* [27], and mouse [28] lipid data.

**Figure 4:**
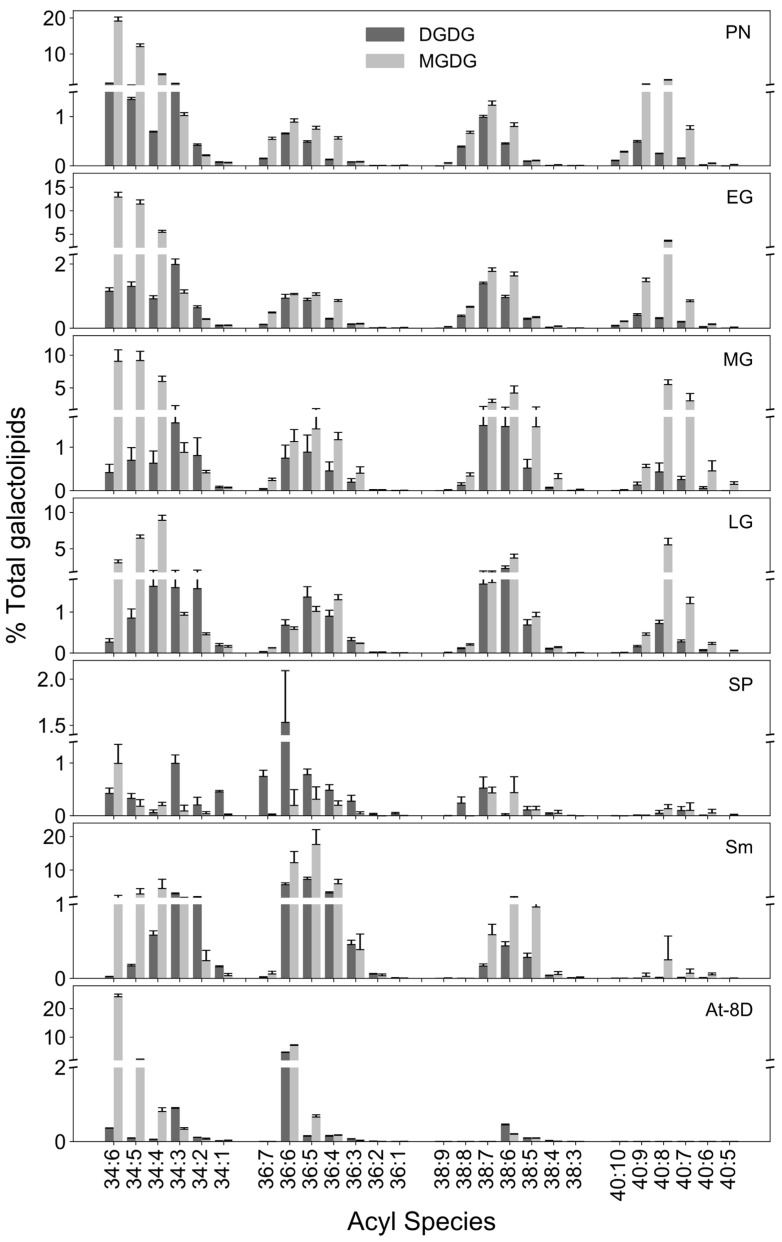
The acyl composition of galactolipids. Acyl composition of MGDG and DGDG in various developmental stages of the moss (protonema (PN), early gametophore (EG), mid-gametophore (MG), late gametophore (LG) and sporophyte (SP) and comparison with Selaginella (Sm) and Arabidopsis (At) 8-day seedlings [27].

Although the early vegetative stages did not vary in their galactolipid content, acyl composition of galactolipids varied with developmental stage. 34C acyl species were prominent in vegetative stages, whereas 36C species were more abundant in sporophytes (Figure 4). Unsaturated long chain acyl species, especially 38C and 40C were more prominent in the moss compared to the vascular plants *Selaginella* and *Arabidopsis* (Figure 4). Major lipid/acyl species for MGDG are 34:6 (18:3/16:3) and 34:5 (18:3/16:2), 34:4 (18:2/16:2), 40:8 (20:4/20:4) 40:9 (20:4/20:5) 40:7 (20:4/20:3), and 38:7 (20:4/18:3) during different developmental stages. In DGDG, 34:3 (18:3/16:2), 34:5 (18:3/16:2), 34:6 (18:3/16:3), 38:6 (20:4/18:2), and 38:7 (20:4/18:3) are dominant. Most of these acyl species composition show similarities to a previous study in *P. patens* [86]. The number of long chains such as 34:6 (18:3/16:3) and 34:5 (18:3/16:2), 34:4 (18:2/16:2) were elevated in early developmental stages compared to the late gametophore and sporophyte stages. Similarly, PUFA levels are higher in protonema than gametophore (Figure 4) [21]. In response to cold, 20:4-containing species was reported to accumulate in *P. patens* glycolipids [9]. In contrast, moss sporophyte had very small amounts of 40C acyl species with 34C, 36C and 38C being the most abundant. 

**Figure 5:**
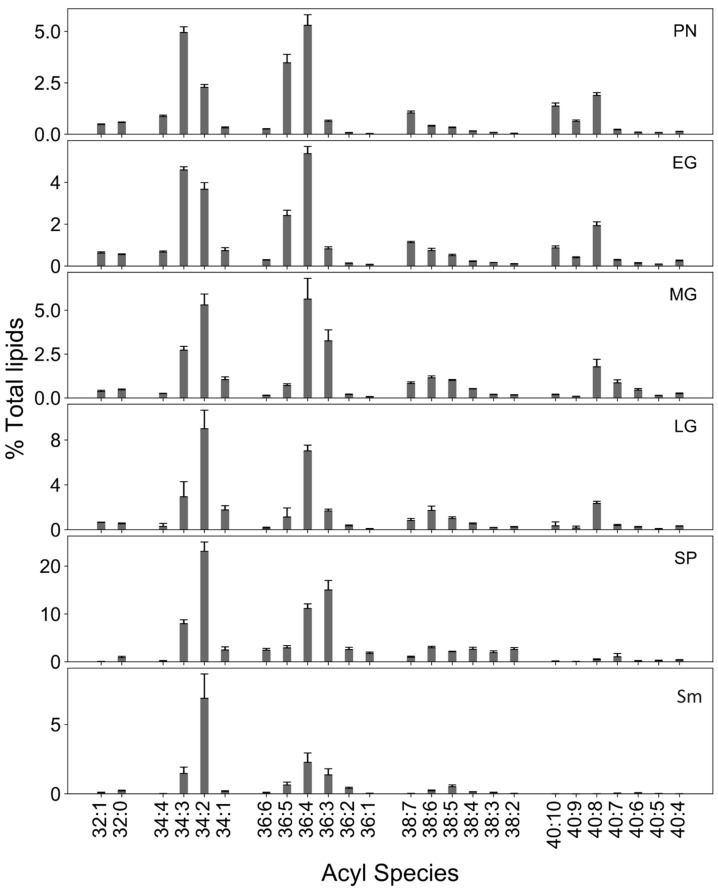
Acyl composition of PC. The acyl composition of various developmental stages (various developmental stages of the moss (protonema (PN), early gametophore (EG), mid-gametophore (MG), late gametophore (LG) and sporophyte (SP)) of the moss, Selaginella (Sm) [27].

Among PC acyl species, 34C (34:3 and 34:2) were the most abundant during moss development, followed by 36C (36:4) (Figure 5). Although 40C species were unique to moss, drastic decreases in levels of 40:8 (20:4/20:4), 40:9 (20:5/20:4), and 40:7 (20:4/20:3) acyl species were observed in sporophyte compared to late gametophore (Figure 5). Similar results were reported during cold stress in *P. patens* where a decrease in PC acyl species, especially those containing 20:4, was observed [9]. In general, 18C and 20C species tend to be polyunsaturated and permit high membrane fluidity that aids in cellular processes such as growth, development, and photosynthesis during adverse conditions. Indeed, an abundance of polyunsaturated 18C and 20C were observed, consistent with high permeability and easy remodeling of the membrane lipids, which may be helpful to plants under environmental stress [87]. However, PC remodeling during transition to sporophyte results in decreased unsaturation and, coupled with a temperature decrease, is likely to increase membrane rigidity. 

**Figure 6:**
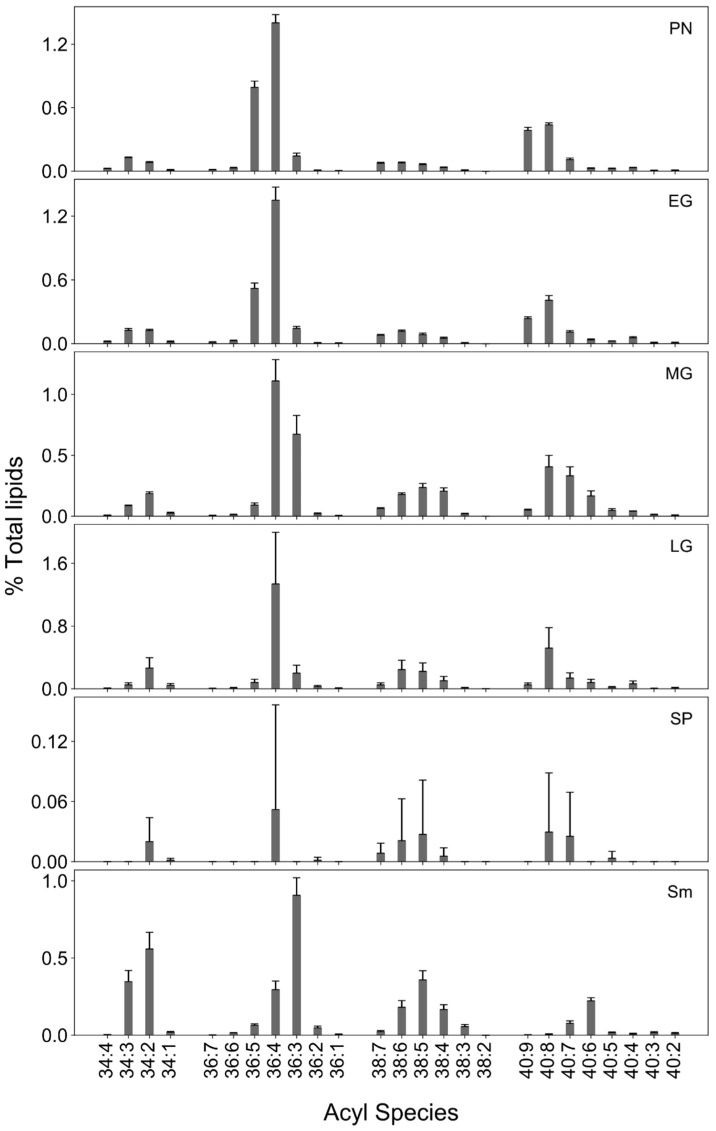
Acyl composition of PE. The acyl composition of PE in developmental stages of the moss (protonema (PN), early gametophore (EG), mid-gametophore (MG), late gametophore (LG) and sporophyte (SP) and comparison with Selaginella (Sm).

Among the PE acyl species, 36C (36:4) was abundant throughout all stages. A gradual shift from 36:5 to 36:3 was observed till the mid-gametophyte (Figure 6). Among 40C acyl species of PE, 40:9 was abundant in protonema and early gametophore although very low in mid-, late-gametophore, and sporophyte. The acyl species 40:9 (20:4/20:5) and 40:8 (mainly 20:4/20:4) comprise 20:4 and 20:5 FA that increase during elevated growth temperatures [7]. The decrease in 40:9 (20:4/20:5) in sporophyte could be due to decrease in temperature. While the presence of very long chain FA in the moss may be responsible for maintaining membrane fluidity and resilience during stress conditions [14,17], 34C species mostly comprise the PE in *Sellaginella* and *Arabidopsis* (Figure 6). 

### 3.6. Abundance of Long-Chain and Polyunsaturated Acyl Species

Long chain FAs are made in plastids and acyl lipids are formed either in plastids or in ER. Extensive FA/lipid transport between the ER and the plastid is important because ER-derived lipids form the intrinsic plastidic membrane. Lipids produced from the prokaryotic pathway contain a large fraction of C16:3 acyl chain because the plant plastid *sn*-2 acyltransferase mostly uses C16-FA as substrate [88]. Lipids produced from the eukaryotic pathway have large fraction of 18:3 acyl chain because ER *sn*-2 acyltransferase has a high specificity for C18-FAs [88]. In the moss, DGDG and MDDG had higher content of 34C, 36C, and 38C acyl species which implies that a large fraction of C18:3 is present (in combination with 16:0 C, 18:1, 18:2) and a desaturase enzyme is involved in C18:1 to 18:3 conversion. Also, in the sporophyte stage, PA consists of acyl chains 34:3 (18:2/16:1), 34:2 (18:2/16:0), 36:4 (18:2/18:2), and 36:3 (18:1/18:2) (Supplementary Table S3). Similarly, an abundance of 18C-FAs was observed in PC and PE as explained earlier (Figure 5 and Figure 6). Minor lipids, PG, PS, PI and lysolipids, were also comprised of higher amounts of 18C and 20C and lower amounts of 16C and 22C (Supplementary Table S3). This indicates that the eukaryotic pathway could be the dominant lipid synthesis pathway in the moss. The presence of long chain FA with a high degree of unsaturation indicates that FAs are produced in the plastid and exported to the ER for lipid assembly via the eukaryotic pathway before the eukaryotic type of glycerol backbone is transported back to the plastid.

### 3.7. Developmental Stages Differ in Double Bond Index and Saturation 

**Figure 7:**
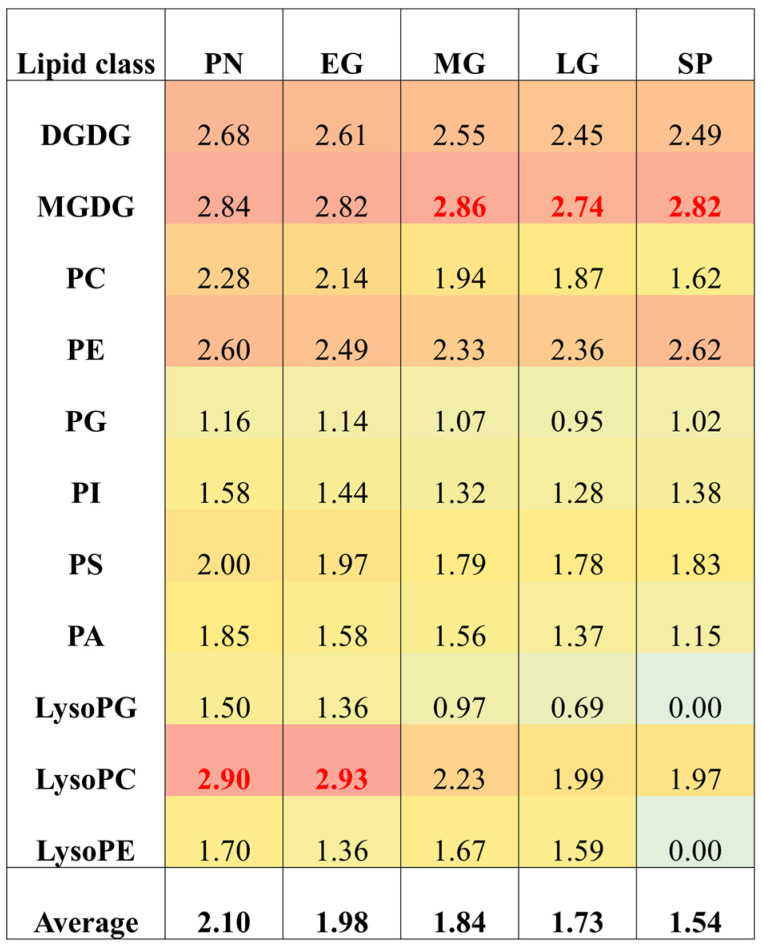
Double bond index (DBI) of major and minor lipid classes during *P. patens* development. The values are shown as heat map with red being the highest, cyan showing the lowest, and yellow representing the mid-range value. The highest DBI values in each growth stages are shown as highlighted red color text. Monogalactosyl diacylglycerol (MGDG), digalactosyl diacylglycerol (DGDG), phosphatidylcholine (PC), phosphatidylethanolamine (PE), phosphatidylglycerol (PG), phosphatidylinositol (PI), phosphatidylserine (PS), phosphatidic acid (PA), lysophosphatidylglycerol (LysoPG), lysophosphatidylcholine (LysoPC), and lysophosphatidylethanolamine (LysoPE).

Double bond index (DBI) measures the number of double bonds in a lipid class. A higher DBI indicates lower saturation (higher unsaturation) and greater membrane permeability. Increased unsaturation generally enhances membrane fluidity. The DBI reflects the average number of double bonds per fatty acid chain. In protonema and early gametophores, the unsaturation of lysoPC is higher compared to other lipid classes, with DBIs of 2.90 and 2.93, respectively. In mid- and late gametophores, and sporophytes, the unsaturation of MGDG is higher (Figure 7). 

Interestingly, the data show that unsaturation generally decreases during moss development, which contrasts with the increase in unsaturation observed during the development of angiosperm leaves as they age. This suggests that the rate of moss growth may exceed the plant’s ability to desaturate lipids, particularly affecting PC, which is the most metabolically active lipid component.

The average DBI for all lipid classes is higher in protonema, with unsaturation levels decreasing as development progresses, reaching their lowest in sporophytes (DBI 1.54). DGDG and MGDG are primarily present in the vegetative stages (Supplementary Table S2), with DBI of DGDG decreasing slightly while that of MGDG remained essentially unchanged. It is unlikely that galactolipids are associated with membrane stability during the transition from vegetative to reproductive stages or in response to temperature stress. Among phospholipids, PC levels are higher in protonema and early gametophore stages compared to later developmental stages. Higher unsaturation of phospholipids, except PE, is observed in early developmental stages. Thus, the higher unsaturation of membrane lipids during early developmental stages is most likely related to moss growth and development. 

## 4. Conclusions

Bryophytes, comprising mosses, liverworts, and hornworts, are ancient land plants with unique evolutionary features that offer valuable insights into plant biology. Despite their significance, the lipid metabolic pathways in bryophytes remain relatively underexplored. This study lays the foundation for investigating these pathways by utilizing -omic analyses to uncover the distinctive aspects of lipid metabolism in these non-vascular plants.

Our research reveals dynamic changes in membrane lipid composition across different developmental stages of the moss. During the vegetative stages, particularly the protonema, there is a high total lipid content and an abundance of VLC-PUFAs, such as 20:4. This lipid profile likely contributes to the moss’s adaptation to cold temperatures and other abiotic stresses by maintaining membrane fluidity. Additionally, the levels of galactolipids, such as MGDG and DGDG, are significantly higher in the vegetative stages compared to the sporophyte stages. The transition to the sporophyte stage is marked by a decrease in galactolipid levels and an increase in phospholipid content, particularly PC. This shift in lipid composition may reflect changes in chloroplast biogenesis and the photosynthetic apparatus during development. Moreover, the increased proportion of PC relative to PE in sporophytes might indicate a protective mechanism against environmental stresses. Additionally, in the sporophyte stage, distinct lipid profile of moss is also characterized by higher percentage of PA. PA is known to accumulate in response to stress, and its elevated levels in sporophytes suggest a potential role in cold temperature adaptation and gametangia formation. These findings underscore the importance of membrane lipid composition in moss development and highlight the lipid adaptations that enable mosses to thrive in diverse environmental conditions.

Future research should address several key questions: How do specific lipid classes contribute to desiccation tolerance and environmental stress responses in bryophytes? Are there novel lipid metabolic pathways in bryophytes that could reveal insights into evolutionary adaptations? How do the lipid metabolic pathways in bryophytes compare to those in vascular plants under similar environmental conditions? Exploring these questions will deepen our understanding of the unique lipid metabolic pathways in bryophytes, providing valuable insights into their adaptation mechanisms and evolutionary strategies. This study enriches our knowledge of plant lipid biology and evolution by uncovering novel biological processes preserved or uniquely evolved in bryophytes.

## Data Availability

The data that support the findings of this study are available from the corresponding author upon reasonable request.

## Supplementary Materials

The following supporting information can be downloaded at: https://www.mdpi.com/article/10.3390/biology13090726/s1, Table S1. Mass spectral acquisition and data processing parameters.; Table S2. Lipid content of major and minor lipid classes during development of the moss (quantified using ESI-MS/MS).; Table S3: Composition of minor lipids; PG, LPG, LPC, LPE, PI, PS and PA during developmental stages of P. patens; protonema (PN), early-, mid- and late- gametophores (EG, MG, LG) and sporophyte (SP). Standard deviations are indicated by “-SD”.
