# Supplementary material for: Dynamic Membrane Lipid Changes in Physcomitrium patens Reveal Developmental and Environmental Adaptations
Source: Biology (Basel). 2024 Sep 16;13(9):726. doi: 10.3390/biology13090726 (PMC11429132; doi:10.3390/biology13090726)
Supplement: Supplementary file 1 [file biology-13-00726-s001.zip › biology-3186625-supplementary.pdf]

Table S1. Mass spectral acquisition and data processing parameters.

Lipid class	Polarity	Scan mode	Fragment mass	Cycles	Declustering potential	Entrance potential	Collision energy	Exit potential	Total mass range
LysoPE, PE	pos	NL	141.02	60	100	15	28	11	420 to 900
LysoPC, PC	pos	Prec	184.07	22	100	14	40	14	450 to 960
MGDG	pos	NL	179.06	75	90	10	21	23	700 to 900
DGDG	pos	NL	341.11	75	90	10	24	23	890 to 1050
PA	pos	NL	115.00	75	100	14	25	14	600 to 850
PG	pos	NL	189.00	75	100	14	20	14	650 to 950
PI	pos	NL	277.00	120	100	14	25	14	790 to 990
PS	pos	NL	185.00	105	100	14	26	14	600 to 920
LysoPG	pos	NL	189.00	150	80	12	25	10	45 to 650

Table S2. Lipid content of major and minor lipid classes during development of the moss (quantified using ESI-MS/MS).

Lipid nmol/mg	PN	EG	MG	LG	SP
MGDG	199.807± 17.848	166.258± 21.066	155.042± 14.242	118.529± 13.496	1.534± 0.555
DGDG	41.128± 3.594	43.954± 8.348	39.034± 22.220	46.266± 13.280	3.144± 0.378
PC	105.683± 8.353	99.447± 15.308	93.979± 26.184	107.094± 14.163	37.510± 3.898
PE	15.523± 2.023	12.886± 1.729	13.274± 2.867	13.629± 1.704	0.079± 0.140
PI	12.634± 1.259	12.027± 3.272	8.948± 6.947	12.296± 5.696	2.988± 2.528
PG	10.731± 16.919	12.622± 11.475	7.515± 6.969	7.298± 5.952	4.706± 1.134
PA	0.334± 0.148	0.467± 0.201	0.428± 0.135	0.746± 0.182	13.460± 2.317
LPC	1.306± 0.991	2.541± 1.617	6.142± 3.294	4.512± 5.258	0.049± 0.040
LPE	0.589± 0.360	0.895± 0.368	1.779± 0.360	1.678± 0.544	0.000± 0.001
LPG	0.444± 0.402	0.657± 0.383	0.351± 0.268	0.235± 0.204	0.000± 0.000
PS	0.537± 0.082	0.529± 0.092	0.503± 0.165	0.526± 0.108	0.039± 0.054
Total lipid	388.715	352.283	326.993	312.808	63.510

Table S3. Composition of minor lipids; PG, LPG, LPC, LPE, PI, PS and PA during developmental stages of P. patens; protonema (PN), early-, mid- and late- gametophores (EG, MG, LG) and sporophyte (SP). Standard deviations are indicated by "-SD".

Compound Name	PN	PN-SD	EG	EG-SD	MG	MG-SD	LG	LG-SD	SP	SP-SD
PG(32:3)	0.029	0.045	0.026	0.035	0.001	0.003	0.000	0.000	0.000	0.000
PG(32:2)	0.027	0.043	0.009	0.009	0.001	0.002	0.000	0.000	0.000	0.000
PG(32:1)	0.234	0.364	0.227	0.197	0.034	0.040	0.029	0.038	0.024	0.048
PG(32:0)	0.415	0.647	0.504	0.425	0.255	0.212	0.226	0.205	2.289	1.020
PG(34:4)	0.606	0.946	0.709	0.626	0.209	0.196	0.088	0.099	0.000	0.000
PG(34:3)	0.704	1.094	0.837	0.729	0.512	0.464	0.348	0.348	1.282	0.470
PG(34:2)	0.442	0.686	0.777	0.652	0.856	0.718	0.839	0.814	4.824	1.389
PG(34:1)	0.110	0.172	0.173	0.152	0.129	0.129	0.249	0.239	0.467	0.275
PG(34:0)	0.001	0.002	0.002	0.005	0.000	0.000	0.000	0.000	0.000	0.000
PG(36:6)	0.000	0.000	0.000	0.001	0.000	0.000	0.000	0.000	0.000	0.000
PG(36:5)	0.003	0.006	0.003	0.003	0.000	0.000	0.000	0.000	0.035	0.041
PG(36:4)	0.013	0.023	0.010	0.013	0.003	0.005	0.009	0.011	1.092	1.070
PG(36:3)	0.000	0.000	0.001	0.002	0.004	0.007	0.000	0.001	1.857	2.146
PG(36:2)	0.000	0.000	0.001	0.002	0.000	0.000	0.000	0.000	0.000	0.000
PG(36:1)	0.000	0.000	0.000	0.000	0.001	0.002	0.000	0.000	0.000	0.000
Total PG	2.585	4.022	3.278	2.811	2.006	1.758	1.788	1.729	11.869	3.224
LPG(16:1)	0.013	0.016	0.006	0.016	0.000	0.000	0.011	0.016	0.000	0.000
LPG(16:0)	0.040	0.068	0.083	0.070	0.054	0.039	0.064	0.071	0.000	0.000
LPG(18:3)	0.038	0.038	0.066	0.069	0.005	0.011	0.004	0.010	0.000	0.000
LPG(18:2)	0.020	0.022	0.017	0.017	0.042	0.032	0.023	0.036	0.000	0.000
LPG(18:1)	0.002	0.006	0.014	0.027	0.000	0.000	0.003	0.008	0.000	0.000
Total LysoPG	0.114	0.102	0.187	0.101	0.101	0.065	0.105	0.096	0.000	0.000
LPC(16:1)	0.006	0.017	0.012	0.039	0.024	0.045	0.023	0.000	0.025	0.000
LPC(16:0)	0.033	0.754	0.080	0.278	0.054	0.681	0.133	0.000	0.544	0.000
LPC(18:3)	0.170	0.181	0.172	0.127	0.249	0.135	0.252	0.001	0.179	0.002
LPC(18:2)	0.012	0.000	0.018	0.274	0.045	0.479	0.070	0.104	0.000	0.085
LPC(18:1)	0.000	0.101	0.000	0.087	0.022	0.157	0.044	0.000	0.141	0.000
LPC(18:0)	0.008	0.020	0.008	0.021	0.013	0.013	0.017	0.000	0.027	0.000
LPC(20:5)	0.067	0.026	0.090	0.008	0.101	0.009	0.102	0.000	0.023	0.000
LPC(20:4)	0.000	0.240	0.000	0.293	0.181	0.394	0.274	0.000	0.624	0.000
LPC(20:3)	0.031	0.098	0.030	0.073	0.052	0.055	0.039	0.000	0.330	0.000
LPC(20:2)	0.003	0.006	0.004	0.019	0.004	0.027	0.006	0.000	0.013	0.000
LPC(20:1)	0.000	0.002	0.000	0.001	0.000	0.002	0.001	0.000	0.003	0.000
LPC(20:0)	0.006	0.020	0.009	0.045	0.031	0.041	0.027	0.002	0.033	0.004
Total LysoPC	0.334	0.946	0.252	1.265	0.776	1.822	0.569	0.107	1.942	0.090
LPE(16:1)	0.000	0.000	0.000	0.000	0.000	0.000	0.000	0.000	0.000	0.000
LPE(16:0)	0.086	0.063	0.046	0.211	0.165	0.145	0.084	0.001	0.232	0.002
LPE(18:3)	0.001	0.005	0.002	0.000	0.001	0.000	0.001	0.000	0.007	0.000
LPE(18:2)	0.002	0.023	0.003	0.060	0.011	0.046	0.009	0.000	0.037	0.000
LPE(18:1)	0.000	0.022	0.001	0.039	0.004	0.044	0.005	0.000	0.057	0.000
LPE(20:5)	0.020	0.003	0.015	0.004	0.012	0.008	0.014	0.000	0.004	0.000
LPE(20:4)	0.031	0.037	0.022	0.120	0.063	0.103	0.047	0.000	0.085	0.000
LPE(20:3)	0.006	0.088	0.007	0.024	0.007	0.032	0.009	0.000	0.142	0.000
LPE(20:2)	0.000	0.015	0.001	0.013	0.002	0.017	0.003	0.000	0.018	0.000
LPE(20:1)	0.000	0.000	0.001	0.000	0.001	0.000	0.002	0.000	0.000	0.000
LPE(20:0)	0.001	0.002	0.003	0.006	0.004	0.009	0.005	0.000	0.001	0.000
Total LysoPE	0.149	0.220	0.089	0.475	0.271	0.340	0.149	0.001	0.582	0.002

PI(32:3)	0.089	0.050	0.094	0.060	0.039	0.028	0.018	0.018	0.000	0.000
PI(32:2)	0.037	0.017	0.012	0.013	0.005	0.007	0.014	0.014	0.000	0.000
PI(32:1)	0.102	0.041	0.133	0.070	0.032	0.030	0.109	0.082	0.002	0.005
PI(32:0)	0.116	0.064	0.150	0.051	0.134	0.084	0.142	0.081	0.027	0.055
PI(34:4)	0.093	0.014	0.069	0.021	0.019	0.016	0.006	0.007	0.000	0.000
PI(34:3)	0.946	0.130	0.794	0.160	0.343	0.230	0.350	0.129	0.273	0.309
PI(34:2)	0.574	0.091	0.918	0.165	1.010	0.750	2.227	0.958	2.429	0.728
PI(34:1)	0.018	0.013	0.071	0.032	0.063	0.047	0.153	0.069	0.109	0.128
PI(36:6)	0.004	0.002	0.002	0.003	0.004	0.005	0.001	0.002	0.074	0.053
PI(36:5)	0.354	0.037	0.231	0.042	0.030	0.020	0.042	0.026	0.176	0.105
PI(36:4)	0.926	0.082	0.878	0.167	0.649	0.478	1.209	0.491	0.437	0.284
PI(36:3)	0.004	0.005	0.014	0.011	0.100	0.088	0.017	0.017	3.486	5.497
PI(36:2)	0.005	0.002	0.002	0.002	0.002	0.002	0.008	0.008	0.036	0.048
PI(36:1)	0.000	0.001	0.001	0.001	0.000	0.001	0.000	0.000	0.000	0.001
Total PI	3.268	0.411	3.369	0.533	2.431	1.710	4.295	1.753	7.050	5.870
PS(34:4)	0.000	0.000	0.000	0.000	0.000	0.000	0.001	0.001	0.000	0.000
PS(34:3)	0.004	0.002	0.005	0.002	0.002	0.001	0.001	0.001	0.000	0.000
PS(34:2)	0.002	0.002	0.004	0.002	0.009	0.002	0.011	0.008	0.003	0.004
PS(34:1)	0.000	0.000	0.000	0.001	0.000	0.001	0.001	0.001	0.002	0.003
PS(36:6)	0.001	0.001	0.000	0.001	0.000	0.001	0.000	0.001	0.007	0.013
PS(36:5)	0.040	0.003	0.036	0.009	0.008	0.006	0.005	0.004	0.000	0.000
PS(36:4)	0.045	0.009	0.061	0.007	0.062	0.010	0.093	0.017	0.033	0.066
PS(36:3)	0.000	0.000	0.000	0.001	0.002	0.002	0.000	0.000	0.000	0.000
PS(36:2)	0.002	0.001	0.001	0.001	0.000	0.000	0.002	0.002	0.000	0.000
PS(36:1)	0.001	0.001	0.000	0.001	0.001	0.001	0.000	0.000	0.000	0.000
PS(38:6)	0.000	0.000	0.000	0.001	0.000	0.000	0.000	0.001	0.000	0.000
PS(38:5)	0.001	0.001	0.001	0.001	0.000	0.001	0.000	0.001	0.000	0.000
PS(38:4)	0.001	0.002	0.002	0.001	0.002	0.003	0.004	0.004	0.000	0.000
PS(38:3)	0.000	0.001	0.001	0.001	0.000	0.000	0.000	0.000	0.008	0.016
PS(38:2)	0.001	0.001	0.001	0.001	0.001	0.002	0.000	0.000	0.000	0.000
PS(38:1)	0.000	0.000	0.000	0.001	0.000	0.000	0.000	0.000	0.000	0.000
PS(40:4)	0.001	0.001	0.002	0.002	0.000	0.000	0.001	0.001	0.000	0.000
PS(40:3)	0.001	0.002	0.000	0.000	0.000	0.000	0.000	0.000	0.000	0.000
PS(40:2)	0.001	0.001	0.000	0.001	0.001	0.001	0.000	0.001	0.000	0.000
PS(40:1)	0.000	0.000	0.001	0.001	0.000	0.000	0.000	0.001	0.000	0.000
PS(42:4)	0.016	0.003	0.013	0.007	0.024	0.006	0.016	0.012	0.018	0.035
PS(42:3)	0.004	0.003	0.002	0.003	0.001	0.001	0.001	0.002	0.000	0.000
PS(42:2)	0.001	0.002	0.002	0.003	0.005	0.004	0.004	0.005	0.001	0.002
PS(42:1)	0.000	0.000	0.000	0.000	0.000	0.000	0.000	0.000	0.000	0.000
PS(44:3)	0.017	0.011	0.019	0.006	0.032	0.018	0.047	0.012	0.023	0.038
PS(44:2)	0.000	0.000	0.000	0.000	0.000	0.000	0.000	0.000	0.000	0.000
Total PS	0.138	0.012	0.151	0.016	0.151	0.020	0.188	0.053	0.094	0.132
PA(32:0)	0.004	0.003	0.006	0.005	0.002	0.005	0.008	0.008	0.000	0.000
PA(34:6)	0.000	0.000	0.000	0.000	0.001	0.001	0.000	0.000	0.000	0.000
PA(34:5)	0.002	0.004	0.000	0.000	0.000	0.000	0.000	0.000	0.000	0.000
PA(34:4)	0.001	0.001	0.001	0.001	0.000	0.000	0.000	0.000	0.000	0.000
PA(34:3)	0.017	0.011	0.021	0.012	0.003	0.006	0.007	0.007	1.718	1.353
PA(34:2)	0.004	0.006	0.034	0.019	0.038	0.015	0.093	0.037	25.437	9.378
PA(34:1)	0.001	0.001	0.001	0.003	0.004	0.005	0.018	0.021	0.148	0.185
PA(36:6)	0.000	0.000	0.000	0.000	0.001	0.001	0.000	0.000	0.000	0.000
PA(36:5)	0.016	0.007	0.012	0.010	0.000	0.001	0.004	0.007	0.000	0.000
PA(36:4)	0.039	0.024	0.050	0.023	0.065	0.012	0.085	0.023	3.346	1.932

PA(36:3)	0.001	0.003	0.005	0.010	0.023	0.030	0.022	0.019	1.721	3.442
PA(36:2)	0.000	0.000	0.001	0.001	0.001	0.002	0.002	0.004	0.000	0.000
Total PA	0.084	0.034	0.131	0.050	0.136	0.049	0.239	0.089	32.370	7.017
